# Foreign Summary

**Published:** 1838-03-14

**Authors:** 


### FOREIGN SUMMARY.

*Case of Atresia Vaginæ.*—In the Journal des Connaissances Medico-Chirurgicales for December, is reported a case of complete occlusion of the vagina, discovered in a woman in labour with her *second* child. This woman had been successfully delivered of a first child; but, owing to erosions in the vaginal parietes from unskilful manipulations, by a midwife, and perhaps to lacerations made by the passage of the child's head, an occlusion appears to have been formed. This was detected by the midwife's finding her finger, in attempting an examination per vaginam, arrested by a cul-de-sac near the middle of that canal. The reporter of the case, Dr. Steinbrenner, being called in, was able to introduce his finger into the vagina, for the distance of two inches and a half, when it encountered a cul-de-sac, without an opening, at the bottom of which he distinguished a sort of raphe resembling a linear citatrix from before backwards, evidently the result of an agglutination of the walls of the vagina. Back of this, a fluctuating mass could be felt, owing to the accumulation of the waters of the amnion, and to the head of the fœtus, which was free in the superior strait. The thickness of the posterior wall of the cul-de-sac seemed to be about two or three lines. The pains being strong and frequent, a slight bleeding was ordered, the rectum emptied by an injection, and it was determined to trust to the powers of nature. Two hours after, as the operator was preparing to practise an incision to effect the delivery, a violent pain supervened, and produced a rupture in the superior extremity of the cicatrix, through which the little finger was introduced and the opening extended. With the index finger all unnatural adherence between the walls of the vagina was broken up, and, in a few minutes, the woman was delivered of a living healthy child. No inflammatory symptom was afterwards manifested in the region of the vagina, and the woman has been since delivered without any similar obstacle, of a third child.

